# Digital Predictors of Morbidity, Hospitalization, and Mortality Among Older Adults: A Systematic Review and Meta-Analysis

**DOI:** 10.3389/fdgth.2020.602093

**Published:** 2021-02-04

**Authors:** Sofia Daniolou, Andreas Rapp, Celina Haase, Alfred Ruppert, Marlene Wittwer, Alessandro Scoccia Pappagallo, Nikolaos Pandis, Reto W. Kressig, Marcello Ienca

**Affiliations:** ^1^Department of Health Sciences and Technology, ETH Zurich, Zurich, Switzerland; ^2^Clever.Care AG, Basel, Switzerland; ^3^FMH Basel, Basel, Switzerland; ^4^Independent Researcher, Tokyo, Japan; ^5^Department of Orthodontics and Dentofacial Orthopedics, School of Dentistry, University of Bern, Bern, Switzerland; ^6^University Department of Geriatric Medicine FELIX PLATTER, Faculty of Medicine, University of Basel, Basel, Switzerland

**Keywords:** digital health (eHealth), systematic (literature) review, meta-analysis, predictor, hospitalization-, mortality, elderly

## Abstract

The widespread adoption of digital health technologies such as smartphone-based mobile applications, wearable activity trackers and Internet of Things systems has rapidly enabled new opportunities for predictive health monitoring. Leveraging digital health tools to track parameters relevant to human health is particularly important for the older segments of the population as old age is associated with *multimorbidity* and higher care needs. In order to assess the potential of these digital health technologies to improve health outcomes, it is paramount to investigate which digitally measurable parameters can effectively improve health outcomes among the elderly population. Currently, there is a lack of systematic evidence on this topic due to the inherent heterogeneity of the digital health domain and the lack of clinical validation of both novel prototypes and marketed devices. For this reason, the aim of the current study is to synthesize and systematically analyse which digitally measurable data may be effectively collected through digital health devices to improve health outcomes for older people. Using a modified PICO process and PRISMA (Preferred Reporting Items for Systematic Reviews and Meta-Analyses) framework, we provide the results of a systematic review and subsequent meta-analysis of digitally measurable predictors of morbidity, hospitalization, and mortality among older adults aged 65 or older. These findings can inform both technology developers and clinicians involved in the design, development and clinical implementation of digital health technologies for elderly citizens.

## Introduction

The growing field of digital health attests that digital technologies are increasingly converging with human health and the delivery of healthcare services. In the last decade, the widespread adoption of, among others, smartphone-based mobile applications, wearable activity trackers, and Internet of Things (IoT) systems, have fuelled a socio-technical trend known as the Quantified Self, i.e., the use of digital technology (broadly defined) for self-tracking purposes (1). Tracking parameters relevant to human health, aiming at improving health outcomes (in short, tracking for health) is a primary justification of self-tracking. The first generation of wearable devices and mobile tools could collect data, and provide insights only related to a small portion of human health and physiology, chiefly mobility reports (e.g., daily steps, physical position). Novel applications have expanded their data sources and can now record a broader variety of health-related parameters and underlying processes. This is due to a four-fold technological transformation. First, self-quantification technologies have expanded in variety as to include data sources that previously could only be collected exclusively via medical devices such as heartbeat rate and electroencephalography (2). Second, smartphone-sensing methods have improved in quality and reliability, now permitting fine-grained, continuous and unobtrusive collection of novel health-related data such as sleep patterns and voice records (3). Third, advances in Artificial Intelligence (AI)-driven software, especially deep learning (4), are increasingly allowing to generate insights about human health from digitally measured data. For example, smartphone apps can be used to predict a person's cognitive status from their responses to gamified cognitive tasks such as 3D virtual navigation (5).

Leveraging digital health to track parameters relevant to human health is particularly important for the older segments of the population as old age is associated with *multimorbidity* (6) and higher care needs. Given the rapid erosion of the old age dependency ratio (reduction in share of working-age people vs. older people) and the often-stated wish of older adults to age in place, these digital technologies can enable novel and more continuous autonomy-preserving tools for health monitoring, prevention and telemedicine. In countries like Italy (34.3%), Switzerland (33.3%), and Germany (32%) this dependency ratio has already shrunk to only three working age people for every person aged 65 and older (7). Personal digital technologies enable continuous and environment-sensitive collection of clinically relevant data which could be used to improve preventative, diagnostic, and therapeutic outcomes. For example, hypertension, systolic, and diastolic blood pressure can be measured by digital sphygmodynamometers and blood pressure monitors. Handheld echo-cardiography can be used for the assessment of a variety of hemodynamic parameters, such as right and left ventricular dimension and function, left ventricular ejection fraction (LVEF), valvulopathies, pulmonary hypertension and arrhythmias. Arrhythmias can also be detected using pulse oximeters, smartwatches, sensors, or contact free electric sensors. ABI can also be measured using portable or digital ABI systems, or automated blood pressure monitors. Diabetes can be measured using a variety of digital blood glucose meters in form of wireless monitors, wearable sensors, or mobile applications. Digital measurements of BMI include digital electronic scales, weight monitors, or smart fat calculators. Respiratory parameters such as respiratory rate, pulmonary ventilation, or oxygen saturation can be measured by pulse oximeters, pressure sensors spirometers, microphones, humidity sensors, accelerometers, or resistive sensors. Finally, physical activity as any other kinematic and cardiovascular factor can be assessed using sensors like patches or necklaces, accelerometers, pedometers, heart rate monitors, or armbands. Balance parameters such as standing, lying, and sitting can be assessed using a variety of sensors, sensitive to capture a wide range of movements in a specific time range. Handgrip strength and muscle strength can be measured using a digital dynamometer. Handgrip strength is also a marker for frailty. Also, a variety of sensors are being used for the diagnosis of fatigue. They are sensitive in detecting circadian variations, electrodermal activity and cardiovascular parameters in fatigue. Furthermore, digital pressure algometers and other devices such as dolorimeters are being used to measure the pressure pain threshold in humans. Finally, for the measurement of fever, new technologies such as wearable thermometers and/or non-contact thermometers have also emerged.

In order to assess the potential of these digital health technologies to improve health outcomes, it is paramount to ground the analysis on solid scientific evidence (8). In particular, it is necessary to investigate which digitally measurable parameters—defined as parameters that are measured or can be measured using personal digital devices—can effectively improve health outcomes among the elderly population. Currently, there is a lack of systematic evidence on this topic due to the inherent heterogeneity of the digital health domain and the lack of clinical validation of both novel prototypes and marketed devices. Our study aims at producing systematic and generalizable knowledge on which digitally measurable data may be effectively collected by future digital health devices to improve health outcomes in certain patient groups. Using a modified PICO process and PRISMA (Preferred Reporting Items for Systematic Reviews and Meta-Analyses) framework (9), we provide the results of a systematic review and subsequent meta-analysis of digitally measurable predictors of morbidity, hospitalization and mortality among older adults aged 65 or older. These findings can inform both technology developers and clinicians involved in the design, development, and clinical implementation of digital health technologies for elderly citizens.

## Methodology

### Search Strategy and Study Selection

**Figure 1:**
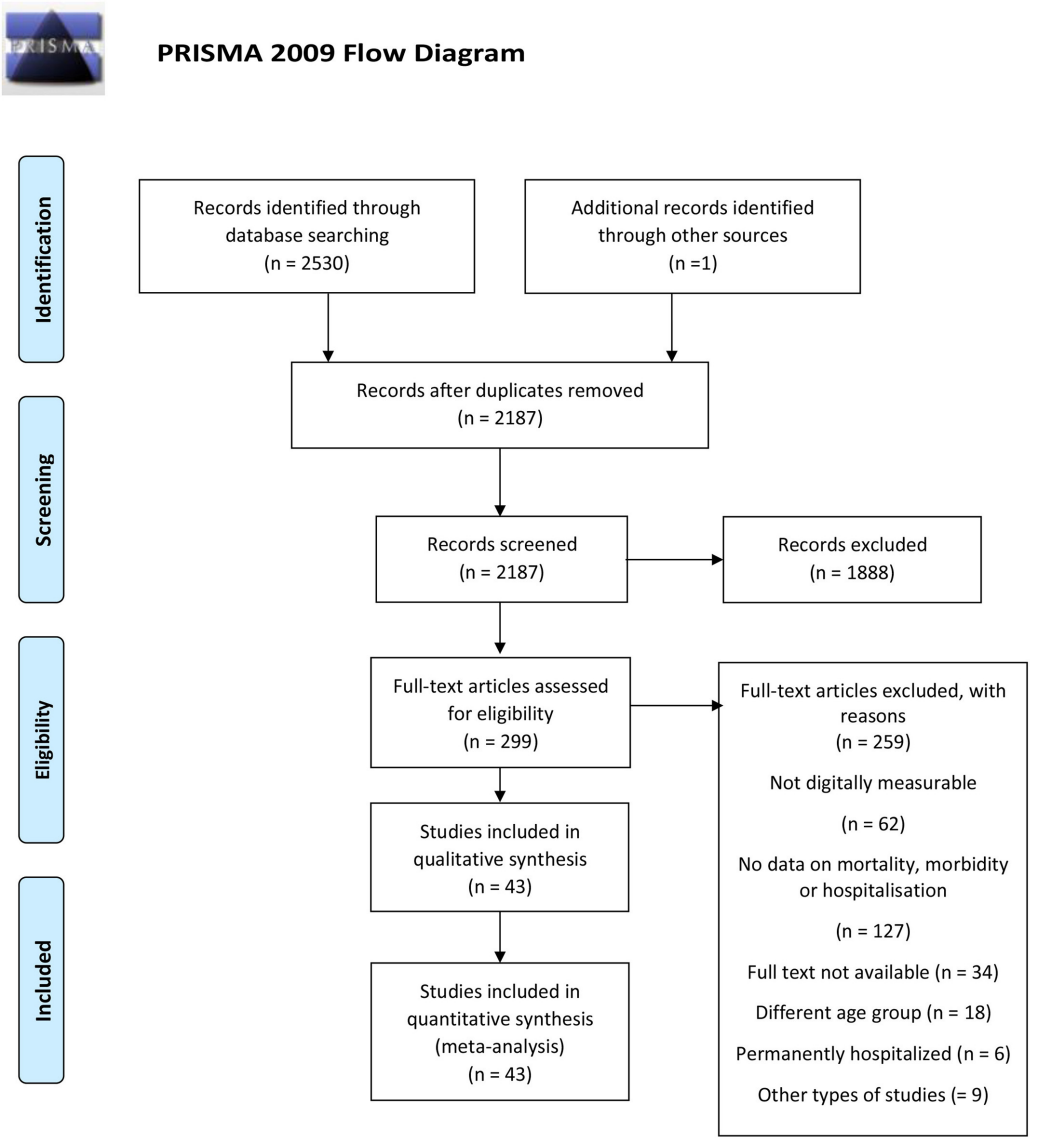
Flowchart.

We searched MEDLINE/Pubmed, Embase, Web of Science and PsycInfo on the 30th of March 2020. We searched the databases for eligible peer-reviewed articles on digitally measurable parameters of hospitalization, morbidity, and mortality published in one of the four languages spoken by the authors, namely English, Italian, Greek, or German. After extensive pilot-testing and validation of the search string, we searched the title, abstract, and keywords using a modified PICO process for studies published from 1995 to 2020 (see Annex 1). We set limitations regarding study type excluding secondary studies (e.g., reviews), theoretical studies and studies with no proof of concept. A full description of the search terms is available as Supplementary Material. A total of 4,266 entries were retrieved using this string. The systematic search was performed by the first author (SD) and inspected for validation by the last author (MI). Query logic was adapted to each search database to optimize retrieval. Following the recommendations by (10), the study selection process was conducted and presented using the Preferred Reporting Items for Systematic Reviews and Meta-Analyses (http://prisma-statement.org/) as a guide (see Figure 1). The PRISMA study selection process entails four phases: identification, screening, eligibility, and final synthesis.

In the screening phase, duplicates were removed both automatically using the Endnote tool for duplicate detection and manually based on abstract screening. A total of 343 articles was removed at this stage. The remaining 2,187 entries were screened manually to remove entries whose ineligibility could be detected via abstract assessments. Thousand eight hundred and eighty-eight records were excluded at this stage. Subsequently, full-text screening was performed on the remaining 299 records. Uncertainties and diverging inclusion choices between the two reviewers were discussed among the research team with documented reasons and re-evaluated until a consensus was reached. Studies included in the synthesis had the features described in Table 1.

**Table 1:** Characteristics of studies included in the final synthesis.

Study type:	Randomized Controlled Trials (RCTs), prospective and retrospective cohort studies.
Population:	Included participants were older people ≥65 years, living in community or residential facilities. Relevant were also studies that included patients temporarily hospitalized for a specific health problem. Studies including people <64 years, residing in hospitals, nursing homes or rehabilitation centers were considered irrelevant.
Outcomes:	Outcomes included all the digitally measurable predictors of mortality and/or morbidity and/or hospitalization events in older people. Studies focusing on implementation issues of digital technology in healthcare or studies aiming to validate the accuracy of different digital devices used in healthcare were excluded. Digital measurements, if available, had to be acquired by digital devices, such as smartphones, robotics, wearable sensors, or medical assistants. Studies including data that were stored in digital databases or electronic medical records and could not be obtained using digital devices, were excluded. Furthermore, studies examining the effectiveness of various approaches or support systems that are available via digital devices such as telemedicine, telehealth, e-prescribing, eLearning, computerized clinical decision support systems (CCDSSs) or computerized provider order entry (CPOE) were also excluded.

### Data Extraction and Coding

We created three different spreadsheets in Microsoft. Excel, one for each of the outcomes reported. Each spreadsheet included information on the study and outcome characteristics (Supplementary Material). Study characteristics included year of publication, study type, sample size, proportion of male participants, mean age, age range, and population diagnoses. For mortality events, we extracted the digitally measurable predictors, the devices used for these measurements and the duration of follow-up period when mortality was measured. For morbidity events, information included all the digitally measurable predictors, all the digital devices used for these measurements and all the adverse health conditions observed after the investigation period. For hospitalization events, coded information included, apart from the digitally measurable predictors and the used devices, the hospital admission and readmission rates. For the estimation of the outcomes we collected all the hazard ratios (HRs), odds ratios (ORs), and 95% Confidence Intervals (CI) reported for mortality, morbidity, and hospitalization events. In cases where the ORs, HRs, and CIs were not provided as primary data by the studies, we calculated them by extracting for each predictor the number of patients with the outcome and the total number of patients for each predictor assigned to each study group (11). For consistency reasons, crude values were preferred over adjusted. We combined HRs and ORs reported separately across studies per gender, per age groups of older people or per quartile of the same predictor across studies, since the aim was an overall outcome assessment without subgroup differentiations (12–17). We also calculated inverse HRs for specific comparisons (18–21).

### Data Analysis

Random-effects meta-analysis was performed using the Knapp-Hartung-Sidik-Jonkman estimator (22, 23). Pooled estimates are presented using odds ratios or hazard ratios. Heterogeneity was assessed using tau2, which defines the variance of the true effects sizes and determines the weight assigned to each of the included studies in the meta-analysis model. In addition, the *I*^2^ statistic which describes the magnitude of heterogeneity across studies that is attributable to the true differences of the results rather than chance or sampling error was also examined (23). Heterogeneity can be interpreted as low, when *I*^2^ = 0–40%, as moderate, when *I*^2^ = 30–60%, as substantial, when *I*^2^ = 50–90% and as considerable when *I*^2^ = 75–100% (24). Meta-regression was performed to examine whether the results differ based on the diagnosis of the participants. The presence of publication bias was assessed using a funnel plot (23, 25). All analyses were performed using Stata 16.1 (StataCorp, TX, USA).

#### Risk of Bias

For RCTs we used the revised Cochrane tool (RoB 2) to assess risk of bias in randomized trials. (26) This tool includes seven items that cover six bias domains; (i) selection bias (2 items); (ii) performance bias; (iii) detection bias; (iv) attrition bias; (v) reporting bias; and (vi) other bias. This tool has three grading levels: (i) low, (ii) moderate, and (iii) high risk of bias. The worst grading in individual items define the overall risk of bias for each single study. For the cohort studies we used the Cochrane Risk Of Bias In Non-randomized Studies—of Interventions (ROBINS-I tool) (27) and also the Newcastle-Ottawa quality assessment scale (NOS) for Cohort Studies. (28) Main domains for risk of the ROBINS-I bias assessment here are: (i) bias due to confounding; (ii) bias in selection of participants into the study, (iii) bias in classification of interventions, (iv) bias due to deviations from intended interventions, (v) bias due to missing data, (vi) bias in measurement of outcomes, and (vii) bias in selection of the reported result. Grading of this scale includes four levels: (i) low, (ii) moderate, (iii) serious, and (iv) critical. Again, the worst grading in any of these items define the overall risk of bias for every single study. The Ottawa scale consists of nine items that cover three dimensions: (i) patient selection (4 items); (ii) comparability of cohorts (2 items); and (iii) assessment of outcome (three items). A point is assigned to each item that is satisfied by the study. The total score therefore ranges from zero to nine, with higher scores indicating higher quality. A total score ≥7 represents high quality.

### Results

A PRISMA flowchart summarizing the article selection process is presented in Figure 1. After the initial database search, 43 studies were considered relevant according to the inclusion criteria and were included in the analysis.

A full description of the included studies is depicted in Tables 2A, 2B. Two of the included studies were RCTs (41, 46), 28 prospective (10, 13–20, 30, 31, 34–40, 43–45, 47, 48, 50, 53, 58–60) and 13 retrospective cohort studies (12, 21, 29, 32, 33, 42, 49, 51, 52, 54–57). The total number of older participants (≥65 years) included in the analysis was 92,994, of whom, 48% (*n* = 44,461) were males and 52% (*n* = 48,533) females. Of the 43 studies, 18 studies included cardiovascular patients, 13 studies included patients with other diagnoses, and in 12 studies the participants had no specific diagnosis. Twenty-nine of the included studies reported results about mortality events, 18 about morbidity and five studies reported results about hospitalization and readmissions. Out of these 43 studies, one study (46) analyzed retrospectively data originating from two independent groups and it was included twice in the analysis. Digital measurements were reported in 15 studies for a wide range of physical and physiological functions. Wearable sensors and stopwatches were used for the measurement of walking speed and other kinematic factors, such as balance parameters. Balance parameters such as standing posture and switches between sitting and standing were also measured by body fixed sensors and stopwatches. Also, a wrist-worn accelometer and an implantable defibrillator were used for the assessment of physical activity. A triaxial wearable gyroscope sensor was used for the measurement of the arterial stiffness and frailty among older people. A digital standing scale was used for the measurement of Body Mass Index (BMI) and an inhome polysomnography for the measurement of respiratory rate. Other devices that were used comprised an automatic device for the Ankle Brachial Index (ABI), an electronic spirometry for the vital capacity, an electric counter for the tapping rate and a digital reactive hyperaemia peripheral arterial tonometry (RH-PAT) for the assessment of the reactive hyperaemia peripheral arterial tonometry index. The remaining 28 studies involved measurements that were not collected using personal digital devices but could have been obtained using commercially available digital devices (e.g., hypertension, systolic and diastolic blood pressure, and arrhythmias as they can be measured via, respectively, digital sphygmodynamometers, blood pressure monitors, and pulse oximeters or smartwatches.

**Table 2A:** Study characteristics—demographic data.

**References**	**Study design**	**Participants *N* (%males), mean age, age range**	**Diagnosis**	**Intervention**
Aboyans et al. (29)	Retrospective cohort	387 (78%), mean age: 68.22 (12.24)	Peripheral arterial disease	Digital Subtraction Angiography
Afilalo et al. (30)	Prospective cohort	131 (66%), mean age: 75.8 (4.4)	Scheduled to undergo cardiac surgery	Cardiac Surgery
Amar et al. (31)	Prospective cohort	100 (74%), mean age: 63.78 (8.48)	No diagnosis	Esophagectomy
Arboix et al. (32)	Retrospective cohort	47 (57%), mean age: 71.55 (10.82)	Thalamic hemorrhage	–
Barnett et al. (33)	Retrospective cohort	8,361 (74.1%), mean age: 63.0 (11.4)	Open heart procedure	Cardiovascular Surgery
Baumert et al. (12)	Retrospective cohort	3,092 (86.9%), mean age: 77.1 (5.65)	No diagnosis/Osteoporosis	–
Berton et al. (34)	Prospective cohort	505 (71.5%), mean age: 68.01	Acute myocardial infraction	Intensive Care Unit
Buch et al. (35)	Prospective cohort	946 (48.8%), age range: 60–80	Age-related macular degeneration	–
Buchman et al. (18)	Prospective cohort	1,249 (23%), mean age: 80.0 (7.72)	No diagnosis	–
Casella et al. (36)	Prospective cohort	1,959 (70%) mean age: 67 (12)	Acute myocardial infraction	Coronary Care Unit
Ferrer et al. (37)	Prospective cohort	328 (38.4%) mean age: 85	No diagnosis	–
Fisher et al. (13)	Prospective cohort	4,910 (43.10%), mean age: 79.4 (5.6)	Age-related macular degeneration	–
Fujishima et al. (38)	Prospective cohort	187 (61%), mean age: 71 (10)	Coronary artery disease	–
Fukumoto et al. (39)	Prospective cohort	1,786 (65.5%), mean age: 65 (10)	Cardiac catheterization	Left-heart Catheterization
Ho et al. (40)	Prospective cohort	1,483 (50.44%), age: >70	No diagnosis	–
Inoue et al. (14)	Prospective cohort	4,373 (0%), mean age: 68.1 (6.6)	No diagnosis	–
Jones et al. (41)	RCT	988 (59%), mean age: 67 (10)	Heart failure	Digitalis vs. Placebo plus Diuretics and Angiotensin-converting–enzyme Inhibitors
Joseph et al., (59)	Prospective cohort	101 (46.5%), mean age: 79 (9)	Patients with ground level fall-injuries	Hospitalized for ground-level falls
Jotheeswaran et al. (10)	Prospective cohort	12,373 (37.7%), mean age: 74.1 (7.0)	Dementia	–
Law et al. (42)	Retrospective cohort	168 (75.6%), mean age: 83.5 (2.5)	Aortic aneurysm	Open and endovascular repair of abdominal aortic aneurysm
Lee et al. (43)	Prospective cohort	1,147 (60.2%), mean age: 64.5 (12.32)	Ischemic stroke	Robotic totally endoscopic coronary artery bypass grafting
Lion et al., (60)	Prospective cohort	61 (0%), mean age: 71.68 (4.28)	No diagnosis	–
Lyyra et al. (15)	Prospective cohort	295 (35.3%), mean age: 75	No diagnosis	–
Matsuzawa et al. (44)	Prospective cohort	140 (0%), mean age: 68.12 (10.58)	Chest pain	Cardiac catheterization
Missouris et al. (45)	Prospective cohort	110 (60%), mean age: 70.8 (9.99)	Peripheral vascular disease	–
Mujib et al. (46)	RCT	7,788 (75%), mean age: 64 (11)	Chronic heart failure	Digoxin or placebo
Oberman et al. (47)	Prospective cohort	3,613 (0%), age range: 50–79	No diagnosis	–
Palmisano et al. (16)	Prospective cohort	770 (66.4%), mean age: 65.5 (14)	No diagnosis	–
Park et al. (48)	Prospective cohort	498 (50%), mea nage: 82.8 (5.3)	30 days phone intervention post hospital discharge	Care transition quality improvement program
Philibert et al. (49)	Retrospective cohort	2,278 (46%), mean age: 66 (9)	No diagnosis	–
Pinto et al. (50)	Prospective cohort	2,968 (48.6), age range: 57–85	Global sensory impairment	–
Plakht et al. (51)	Retrospective cohort	2,763 (67.8%), mean age: 66.6 (13.3)	Acute myocardial infraction	Hospitalization after atrial myocardial infraction
Radhakrishnan et al. (52)	Retrospective cohort	403 (45%), age range: <60–>94	No diagnosis	Telehealth
Rolland et al. (17)	Prospective cohort	7,250 (0%), mean age: 80.5 (3.76)	Hip fracture	–
Sheng et al. (53)	Prospective cohort	4,055 (44.5%), mean age: 68.5 (7.5)	Peripheral arterial disease	–
Smirnova et al. (54)	Retrospective cohort	2,978 (51%), mean age: 65.9 (9.6)	No diagnosis	–
Stein et al. (19)	Prospective cohort	1,655 (82%), mean age: 66.81 (11.69)	ICD implantation	Implantable cardioverter defibrillator
Tateishi et al. (55)	Retrospective cohort	164 (53%), median age: 76	Acute ischemic stroke	Thrombolysis.
van Vugt et al. (56)	Retrospective cohort	1,614 (55.5%), mean age: 68.6 (10.8)	Colorectal cancer	Colorectal cancer surgery
Ward et al. (57)	Retrospective cohort	309 (43.3%), mean age: 65.7 age range: 34–94	Peripheral arterial disease	–
Wilson et al. (58)	Prospective cohort	5,630 (35.1%), age: >70	No diagnosis	–
Zeitzer et al. (20)	Prospective cohort	2,976 (100%), mean age: 76.4 (5.53)	Osteoporosis	–
Zhu et al. (21)	Retrospective cohort	186 (84.4%), mean age: 65	Bladder cancer	Robot-assisted radical cystectomy

**Table 2B:** Study characteristics—predictors and outcomes.

**References**	**Predictor type (digitally measurable)**	**Digital device used**	**Outcome**	**Time spectrum for mortality**	**Morbidity severity**	**Hospital admission**
Aboyans et al. (29)	Hypertension, diabetes	No digital device mentioned	Mortality, morbidity, hospitalization	62 months	Renal artery stenosis	Intensive Care Unit admission
Afilalo et al. (30)	Slow walking speed	Stopwatch	Morbidity	–	Morbidity	–
	Left ventricular ejection fraction <40		Mortality, morbidity			
Amar et al. (31)	Supraventricular tachydysrhythmias	No digital device mentioned	Mortality	30 days	Supraventricular tachydysrhythm-ias	–
	Hypertension, diabetes	No digital device mentioned	Morbidity			
Arboix et al. (32)	Respiratory events, obesity	No digital device mentioned	Mortality	Not mentioned	–	–
Barnett et al. (33)	Hypertension, diabetes, ejection fraction >40, obesity	No digital device mentioned	Mortality	30 days	–	–
Baumert et al. (12)	Respiratory rate ≥16 breaths·min 1	Overnight in-home Polysomnography	Mortality	6.4 (1.6) years /8.9 (2.6) years	–	–
Berton et al. (34)	Diabetes	No digital device mentioned	Mortality, morbidity	–	Atrial fibrillation/ flutter	–
	Hypertension, arrhythmias	No digital device mentioned				
			Morbidity			
Buch et al. (35)	Hypertension, diabetes	No digital device mentioned	Mortality	14 years	–	–
Buchman et al. (18)	Standing posture, Timed Up and Go (TUG) stand, TUG sit to stand, TUG stand to sit, 32-ft. Slow walking speed	Body-fixed sensor	Mortality, morbidity	3.6 years	Mild cognitive impairment, Dementia	–
Casella et al. (36)	Systolic blood pressure ≤90, diabetes, heart rate >100 b/min	No digital device mentioned	Mortality	30 days	–	–
Ferrer et al. (37)	Atrial fibrillation	No digital device mentioned	Mortality	3 years	–	–
Fisher et al. (13)	Decreased BMI, increased BMI, hypertension, diabetes	No digital device mentioned	Mortality	4.8 years	–	–
Fujishima et al. (38)	Hypertension, diabetes, diastolic blood pressure, increased BMI	No digital device mentioned	Morbidity	–	Ischemia	–
Fukumoto et al. (39)	Hypertension, diabetes, atrial fibrillation, left ventricular ejection fraction <45	No digital device mentioned	Morbidity	–	Cholesterol embolization syndrome	–
Ho et al. (40)	Hypertension, diabetes	No digital device mentioned	Morbidity	–	Mobility decline	–
	Decreased BMI, increased BMI,	Digital standing scale				
	Slow walking speed	Stopwatch				
Inoue et al. (14)	Arterial stiffness	Triaxial wearable gyroscope sensor	Mortality	9 years	–	–
Jones et al. (41)	Decreased BMI, increased BMI, diabetes, ejection fraction	No digital device mentioned	Mortality	3.5 years	–	–
Joseph et al., (59)	Upper-extremity function-frailty	Triaxial wearable gyroscope sensor	Hospitalization	–	–	Institutionalization, readmissions
Jotheeswaran et al. (10)	Weight loss, decreased physical activity, slow walking speed	Standard timed walking test for walking speed	Mortality	2.8–5 years	Incident dependence	–
	Slow walking speed		Morbidity			
Law et al. (42)	Hypertension, diabetes	No digital device mentioned	Mortality	30 days	–	–
Lee et al. (43)	Abnormal ABI <0.90	Automatic device	Mortality	30 days	–	–
Lion et al., (60)	Systolic blood pressure	No digital device mentioned	Morbidity	–	Dizziness	–
Lyyra et al. (15)	Vital capacity	Electronic spirometer	Mortality	10 years	-	–
	Tapping rate	Electric counter				
	Muscle strength	Not mentioned				
	Slow walking speed	Stopwatch				
Matsuzawa et al. (44)	Reactive hyperaemia peripheral arterial tonometry index	Digital reactive hyperaemia peripheral arterial tonometry (RH-PAT)	Morbidity	–	Ischemic heart disease	–
	Diabetes, systolic blood pressure, diastolic blood pressure, increased BMI	No digital device mentioned				
Missouris et al. (45)	Hypertension, diabetes	Semi-automated ultrasound sphygmomanometer	Mortality	6.1 years	–	–
Mujib et al. (46)	Hypertension, diabetes, ejection fraction <35	No digital device mentioned	Morbidity	–	Stroke/neurological deficit	–
Oberman et al. (47)	Hypertension, diabetes, systolic blood pressure	No digital device mentioned	Morbidity	–	Hypertrophied left ventricular mass	–
Palmisano et al. (16)	Decreased physical activity	Implantable cardioverter defibrillator	Morbidity	–	Atrial arrhythmias	–
Park et al. (48)	Weakness, pain, palpitations, fatigue, fever, weight gain, difficulty breathing	No digital device mentioned	Hospitalization	–	–	30-day readmissions
Philibert et al. (49)	Systolic blood pressure, diastolic blood pressure, diabetes	No digital device mentioned	Mortality	Not mentioned	–	–
Pinto et al. (50)	Hypertension, diabetes, increased BMI, decreased BMI, obesity	No digital device mentioned	Morbidity	–	Impaired mobility	–
Plakht et al. (51)	Hypertension, obesity, atrial fibrillation, severe left ventricular dysfunction, significant left ventricular hypertrophy, left ventricular dilatation, left ventricular filling pressure, moderate or severe mitral valve regurgitation pulmonary hypertension, left atrial dilatation, right ventricular dysfunction, moderate or severe tricuspid regurgitation	No digital device mentioned	Mortality	8.2 years	–	–
Radhakrishnan et al. (52)	Pulmonary and arrhythmia comorbidity, obesity	No digital device mentioned	Hospitalization	–	–	Admission
Rolland et al. (17)	Slow walking speed, repeated chair stands, balance test	Stopwatch	Mortality	3.8 years	–	–
	Low handgrip strength	Not mentioned				
Sheng et al. (53)	Hypertension, diabetes	Blood pressure monitor	Mortality	5.9 years	–	–
Smirnova et al. (54)	Obesity, diabetes	No digital device mentioned	Mortality	5 years	–	–
Stein et al. (19)	Atrial fibrillation, diabetes, decreased BMI, obesity, left ventricular ejection fraction <40, increased physical activity, decreased physical activity	No digital device mentioned	Mortality	1 year	–	–
Tateishi et al. (55)	Hypertension, diabetes	No digital device mentioned	Mortality, morbidity	90 days	Unfavorable outcome	–
	Atrial fibrillation		Morbidity			
van Vugt et al. (56)	Increased BMI, decreased BMI, obesity	No digital device mentioned	Mortality, hospitalization	30 days	–	LOS, readmission
Ward et al. (57)	Hypertension, diabetes, abnormal ABI <0.90	No digital device mentioned	Morbidity	–	Left ventricular ejection fraction <35	–
Wilson et al. (58)	Hypertension, increased BMI, decreased BMI, walking difficulty/slow walking speed	No digital device mentioned	Morbidity	–	Hip fracture	–
Zeitzer et al. (20)	Increased physical activity, decreased physical activity	Wrist-worn accelerometer	Mortality	6.5 years	–	–
Zhu et al. (21)	Increased BMI	No digital device mentioned	Mortality	Not mentioned	–	–

### Risk of Bias Assessment

Risk of bias assessment was performed independently by two authors (Tables 3–5). Disagreements were solved through discussion and re-evaluation of the differently evaluated points until a consensus was reached. According to RoB and ROBINS-I scales, 17 studies were assessed as being of serious risk of bias, 16 studies were assessed of moderate risk of bias and only 10 studies were assessed as being of low risk of bias. According to the Newcastle Ottawa Scale, only six studies had a total score ≥ 7.

**Table 3:** Additional predictors of Mortality.

**No**.	**References**	**Predictor**	**HR/OR (95% CI)**	***p***
1.	Arboix et al. (32)	Respiratory events	OR 3.30; CI 0.62, 17.62	0.081
2.	Baumert et al. (12)	Respiratory rate ≥16 breaths·min^−1^	HR 1.44; CI 1.29, 1.62	<0.00001
3.	Inoue et al. (14)	Arterial stiffness	HR 2.98; CI 2.08, 4.27	<0.00001
3.	Lee, (43)	ABI>90	HR 2.33; CI 1.24, 4.39	0.009
5.	Lyyra (15)	Vital capacity	HR 0.07; CI 0.03, 0.19	<0.00001
6.	Lyyra (15)	Tapping rate	HR 0.04; CI 0.77, 0.91	<0.00001
7.	Lyyra (15)	Muscle strength	HR 0.73; CI 0.58, 0.92	0.007
8.	Philibert et al. (49)	Diastolic Blood Pressure	HR 0.67; CI 0.60, 0.75	<0.0001
9.	Plakht et al. (51)	Severe Left ventricular dysfunction	HR 2.12; CI 1.75; 2.57	0.002
10.	Plakht et al. (51)	Concentric or significant left ventricular hypertrophy	HR 1.96; CI 1.49; 2.57	0.788
11.	Plakht et al. (51)	Left ventricular dilatation	HR 1.79; CI 1.3; 2.47	0.003
12.	Plakht et al. (51)	Left ventricular filling pressure	HR 1.17; CI 1.09; 1.24	0.152
13.	Plakht et al. (51)	Moderate or severe mitral valve regurgitation	HR 1.47; CI 1.14; 1.9	<0.001
14.	Plakht et al. (51)	Moderate or severe pulmonary hypertension	HR 1.88; CI 1.48; 2.39	0.001
15.	Plakht et al. (51)	Left atrial dilatation	HR 1.24; CI 1.14; 1.35	0.929
16.	Plakht et al. (51)	Right ventricular dysfunction	HR 1.22; CI 1.1; 1.36	0.159
17.	Plakht et al. (51)	Moderate or severe tricuspid regurgitation	HR 1.65; CI 1.29; 2.1	<0.001
18.	Rolland et al. (17)	Low handgrip strength	HR 1.47; CI 1.18, 1.83	0.0006

**Table 4:** Additional predictors of Morbidity.

**No**.	**References**	**Predictor**	**OR/HR; (95% CI)**	***p***
1.	Buchman et al. (18)	Slow walking speed for MCI	HR 115; CI 0.93, 1.42	0.08
2.	Buchman et al. (18)	Slow walking speed for Dementia	HR 1.62; CI 1.23, 2.14	0.0006
3.	Jotheeswaran et al. (10)	Slow walking speed	IRR 1.28; CI 1.12, 1.47	0.003
4.	Matsuzawa et al. (44)	Reactive hyperemia peripheral arterial tonometry index	OR 0.51; CI 0.38, 0.66	<0.00001
5.	Palmisano et al. (16)	Decreased physical activity	OR 5.56; CI 2.45, 12.64	<0.0001
6.	Pinto et al. (50)	BMI obesity	OR 1.12; CI 0.76, 1.63	0.57
7.	Ward et al. (57)	ABI <0.90	OR 2.48; CI 1.22, 5.07	0.01

**Table 5:** Predictors of hospitalization.

	**No**.	**References**	**Predictor type (digitally measurable)**	**OR (95% CI)**	***p***
Hospital admission	1.	Joseph, (59)	Upper-Extremity Function-Frailty	OR 4.14; CI 1.63, 10.51	0.000
	2.	Radhakrishnan et al. (52)	Pulmonary comorbidity	OR 3.43; CI 1.12, 10.5	0.031
			Arrhythmia comorbidity	OR 0.60; CI 0.36, 1.0	0.051
			Obesity	OR 2.69; CI 1.0, 7.19	0.048
Hospital readmission	1.	Joseph, (59)	Upper-Extremity Function-Frailty	OR 2.12; CI 0.89, 5.16	0.045
	2.		Upper-Extremity Function-Frailty	OR 2.37; CI 1.05, 5.33	0.019
	3.	Park et al. (48)	Weakness	OR 0.68; CI 0.42, 1.09	0.055
	4.		Pain	OR 0.42; CI 0.21, 0.85	0.008
	5.		Palpitations	OR 1.62; CI 0.6, 4.38	0.172
	6.		Fatigue	OR 3.04; CI 1.15, 8.03	0.013
	7.		Fever	OR 1.15; CI 0.14, 9.33	0.045
	8.		Weight gain	OR 3.83; CI 1.30, 11.27	0.007
	9.		Difficulty breathing	OR 1.58; CI 0.68, 3.64	0.144
	10.	van Vugt et al. (56)	Increased BMI	OR 0.91; CI 0.66, 1.26	0.291
	11.		Decreased BMI	OR 0.95; CI 0.46, 1.97	0.441
	12.		Obesity	OR 1.03; CI 0.68, 1.58	0.044

## Results

### Mortality

**Figure 2:**
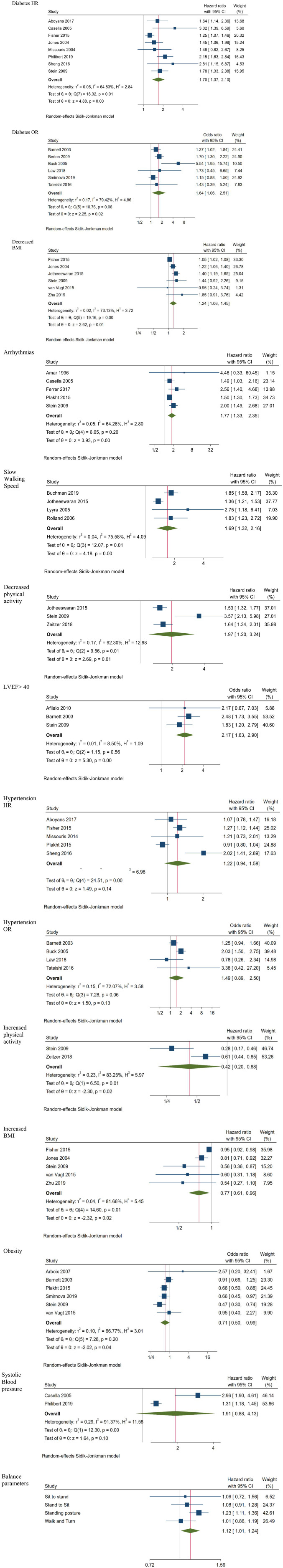
Forest plots for predictors of Mortality.

Meta-analysis of all the digitally measurable predictors of mortality identified by the search, indicated six statistically significant predictors (Figure 2). These included diabetes (HR 1.70; CI 1.37, 2.10; 8 studies; OR 1.64; CI 1.06, 2.51; 6 studies), decreased BMI (HR 1.24; CI 1.06, 1.45; 6 studies), arrhythmias (HR 1.77; CI 1.33, 2.35; 5 studies), slow walking speed (HR 1.69; CI 1.32, 2.16; 4 studies), not being physically active (HR 1.97; CI 1.20, 3.24; 3 studies) and LVEF <40 (OR 2.17; CI 1.63, 2.90; 3 studies). Hypertension results were marginally insignificant for mortality (HR 1.22; CI: 0.94, 1.58; 5 studies; OR 1.49; CI: 0.89, 2.50; 4 studies). Being physically active, having increased BMI and obesity were significantly associated with survival (HR 0.42; CI 0.20, 0.88; 2 studies, HR 0.77; CI 0.61, 0.96; 5 studies and OR 0.71; CI 0.50, 0.99; 6 studies, respectively), whereas the results related to systolic blood pressure ≤90 (HR 1.91; CI 0.88, 4.13; 2 studies) were neither for mortality nor for survival statistically significant.

The description of four balance parameters was based on two studies on standing posture [HR 1.23; CI 1.11, 1.36; (18) and (17)] and sit-to-stand ability [HR 1.06; CI 0.72, 1.56; (18) and (17)] while measurements of the other two variables were reported only in one study (18). Since these multiple balance measurements were originating from the same samples, we did not estimate a common effect size for all the balance parameters to avoid the unit of analysis error (23). In contrast, we created a forest plot visual representation of the outcomes which indicates that the only significant predictor of mortality was pooled standing posture (HR 1.23; CI 1.11, 1.36; 2 studies).

Table 3 summarizes additional predictors of mortality identified only once across the included studies. According to these results, a respiratory rate ≥16 breaths·min^−1^ (HR 1.44; CI 1.29, 1.62), arterial stiffness (HR 2.98; CI 2.08, 4.27), ABI >90 (HR 2.33; CI 1.24, 4.39) severe left ventricular dysfunction (HR 2.12; CI 1.75; 2.57) significant left ventricular hypertrophy (HR 1.96; CI 1.49; 2.57), left ventricular dilatation (HR 1.79; CI 1.3; 2.47), left ventricular filling pressure (HR 1.17; CI 1.09; 1.24), moderate or severe mitral valve regurgitation (HR 1.47; CI 1.14; 1.9), pulmonary hypertension (HR 1.88; CI 1.48; 2.39), left atrial dilatation (HR 1.24; CI 1.14; 1.35) right ventricular dysfunction (HR 1.22; CI 1.1; 1.36), moderate or severe tricuspid regurgitation (HR 1.65; CI 1.29; 2.1), and low handgrip strength (HR 1.47; CI 1.18, 1.83) were significantly associated to mortality. Vital capacity (HR 0.07; CI 0.03, 0.19), high tapping rate (HR 0.04; CI 0.77, 0.91), and muscle strength (HR 0.73; CI 0.58, 0.92), were significant predictors of longer survival.

Current analysis represents a synthesis of the digitally measurable predictors of mortality. The analysis indicates that a variety of crucial health-related survival parameters, such as hemodynamic, respiratory, kinetic measurements, BMI and diabetes, can be measured and managed remotely. Digital technologies such as blood pressure monitors, pulse oximeters, and sensors for the measurement of heart and respiratory rate, blood glucose meters for diabetes, height-weight monitors for BMI, movement sensors, accelerometers, pedometers for physical activity parameters, dynamometers for muscle strength, spirometers, and hand-held echocardiogram can be efficiently incorporated in routine-care of older people, since they are correlated with survival or mortality, respectively.

Subgroup analyses comparing participants with cardiovascular diseases to those with no cardiovascular diagnoses were performed on three of the statistically significant predictors of mortality. Subgroup analysis for diabetes (HR 1.69; CI 1.43, 2.00 for cardiovascular vs. HR 1.62; CI 0.95, 2.75 for other diagnoses) and decreased BMI (HR 1.24; CI 1.08, 1.42 for cardiovascular patients vs. HR 1.24; CI 0.96, 1.61 for other diagnoses) indicated that diabetes and decreased BMI are significant predictors of mortality only for cardiovascular patients, whereas arrhythmias (HR 1.61; CI 1.35, 1.93 for cardiovascular vs. HR 2.63; CI 1.46, 4.74 for other diagnoses) did not differentiate across diagnoses regarding their association with mortality.

We did not perform a subgroup analysis for slow walking speed, not being physically active and LVEF <40 and since, in the first case none of the studies included cardiovascular patients, and in the two last cases the number of studies included was not sufficient for a subgroup analysis.

Some of the previous analyses were based on a small number of studies and the instability of these results should be considered.

The 95% Confidence Intervals of the included studies are very narrow, and although estimates are close to each other suggesting homogeneity, the *I*^2^ is relatively high (23, 24). Non-significant heterogeneity tests for Hypertension in ORs (*I*^2^ = 59, *p* = 0.06) and systolic blood pressure (*I*^2^ = 92, *p* = 0.11) possibly occurred due to low power, since the number of studies included in these analyses was small (23).

### Morbidity

**Figure 3:**
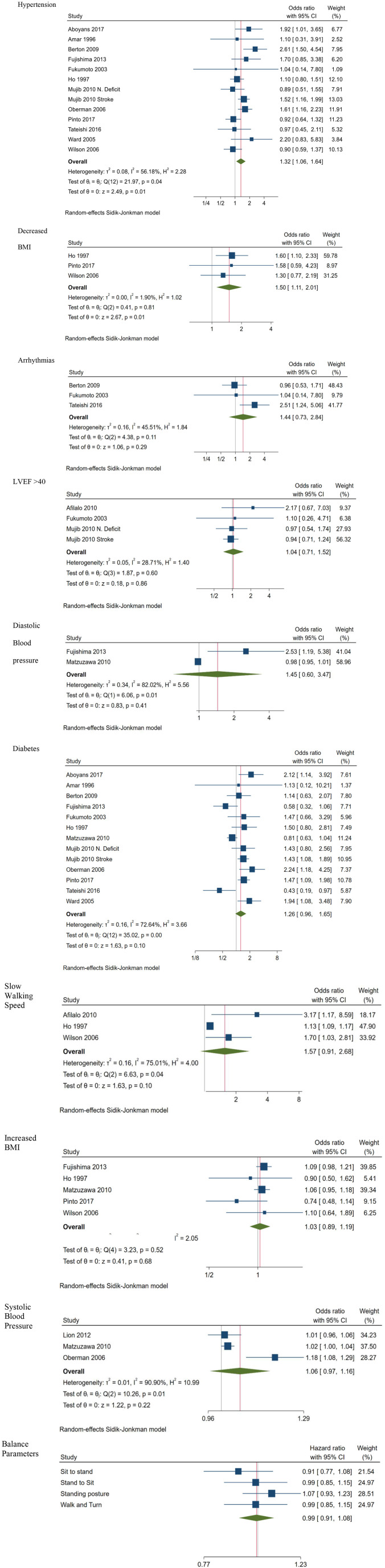
Forest plots: predictors of Morbidity.

Predictors of morbidity are depicted in Figure 3. Hypertension (OR 1.32; CI 1.06, 1.64; 13 studies), and decreased BMI (OR 1.50; CI 1.11, 2.01; 3 studies) were identified as significant predictors of morbidity. Meta-analysis of outcomes reporting results for arrhythmias (OR 1.44; CI 0.73, 2.84; 3 studies), LVEF <45 (OR 1.04; CI 0.71, 1.52; 4 studies) and diastolic blood pressure (OR 1.45; CI 0.60, 3.47; 2 studies) did not provide any statistically significant result, whereas results regarding diabetes (OR; CI 1.26 0.96, 1.65; 13 studies), slow walking speed (OR 1.57; CI 0.91, 2.68; 3 studies) increased BMI (OR 1.03; CI 0.89, 1.19; 5 studies) and systolic blood pressure (OR 1.06; CI 0.97, 1.16; 3 studies) were marginally insignificant.

Figure 3 also provides a visualization of the contribution of each of the four balance parameters to morbidity, based on the combined outcomes of two independent groups originating from the same study (18). Results indicate that none of them was associated to dementia and/or mild cognitive impairment or to being healthy.

Other statistically important predictors of morbidity identified only once through the literature search (Table 4), were slow walking speed reported as HR for dementia (HR 1.62; 1.23, 2.14) and as incidence rate ratio IRR (IRR 1.28; 1.12, 1.47). Other statistically important predictors of morbidity were decreased physical activity (OR 5.56; CI 2.45, 12.64) and an abnormal ABI <90 (OR 2.48; CI 1.22, 5.07).

Subgroup analysis indicated that hypertension was a significant predictor of morbidity for cardiovascular patients, compared to people with other diagnoses (OR 1.55; CI 1.19, 2.00 vs. OR 1.12; CI 0.88, 1.43), while diabetes was a significant predictor of morbidity only for non-cardiovascular patients (OR 1.23; CI 0.95, 1.59; for cardiovascular vs. OR 1.57; CI 1.22, 2.00 for other diagnoses).

Heterogeneity was moderate for hypertension (*I*^2^ = 45, *p* = 0.006) and diabetes (*I*^2^ = 47, *p* = 0.004), and no heterogeneity was evident for decreased BMI studies (*I*^2^ = 0 *p* = 0.006). The small number of studies included in the remaining analyses, could account for the non-significant heterogeneity values, indicating limiting power for estimating the true effect (23).

### Hospitalization

Two studies reported results about predictors of hospitalization, three about predictors of hospital readmission and one study provided a ratio for the Intensive Care Unit (ICU) admission. Identified predictors of the included studies are presented in Table 5. The odds for hospitalization were higher for people with ground-level fall injuries diagnosed as frail compared to those who were not diagnosed as frail (OR 4.14; CI 1.63, 10.51). Obesity (OR 2.69; CI 1.0, 7.19) and pulmonary problems (OR 3.43; CI 1.12, 10.5) were significant predictors of hospitalization for people with colorectal cancer. Frailty (OR 2.37; CI 1.05, 5.33) was reported as a significant predictor of 60-day readmission for people with fall injuries, while fatigue (OR 3.04; CI 1.15, 8.03) and weight gain (OR 3.83; CI 1.30, 11.27) were reported as significant predictors of 30-day readmissions for people with a history of hospitalization followed for 30 days after the last hospital admission. Finally, supraventricular tachydysrhythmias seem to be an important predictor of ICU admission (OR 18.9; CI 4.59, 77.87) for people that have undergone esophageal operation. Although hospitalization outcomes did not provide us with an adequate number of studies to proceed to an analysis with multiple predictors, we succeeded however to find associations between additional technologies and health management of older people. These technologies include digital dynamometers for the assessment of frailty and weakness in older people, sensors, sensitive in identifying fatigue symptoms and, digital pressure algometers and dolorimeters for the measurement of pain.

### Limitations

**Figure 4:**
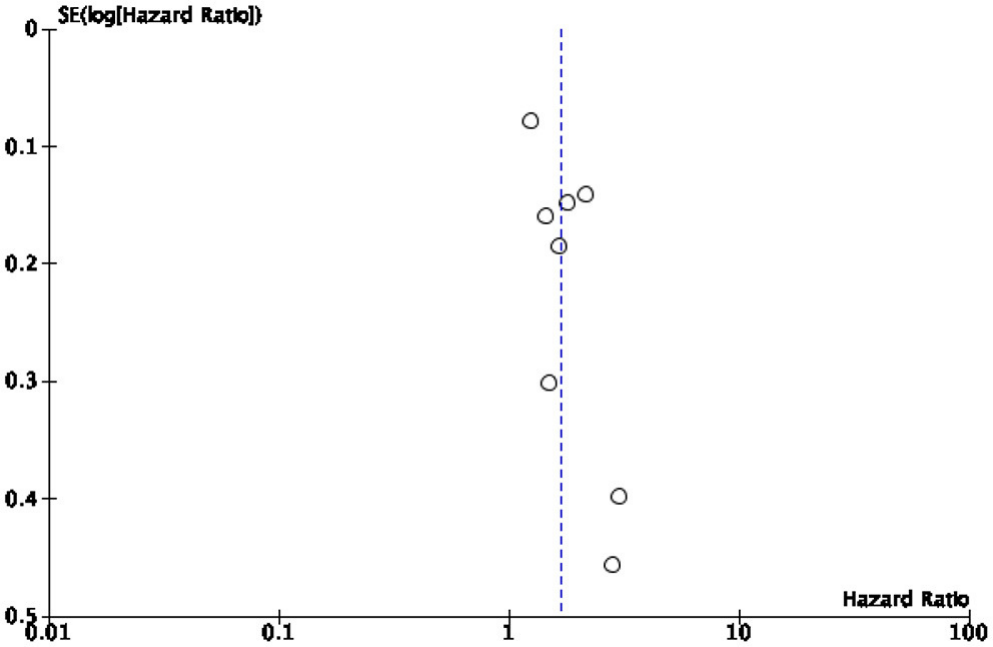
Publication bias for Mortality.

**Figure 5:**
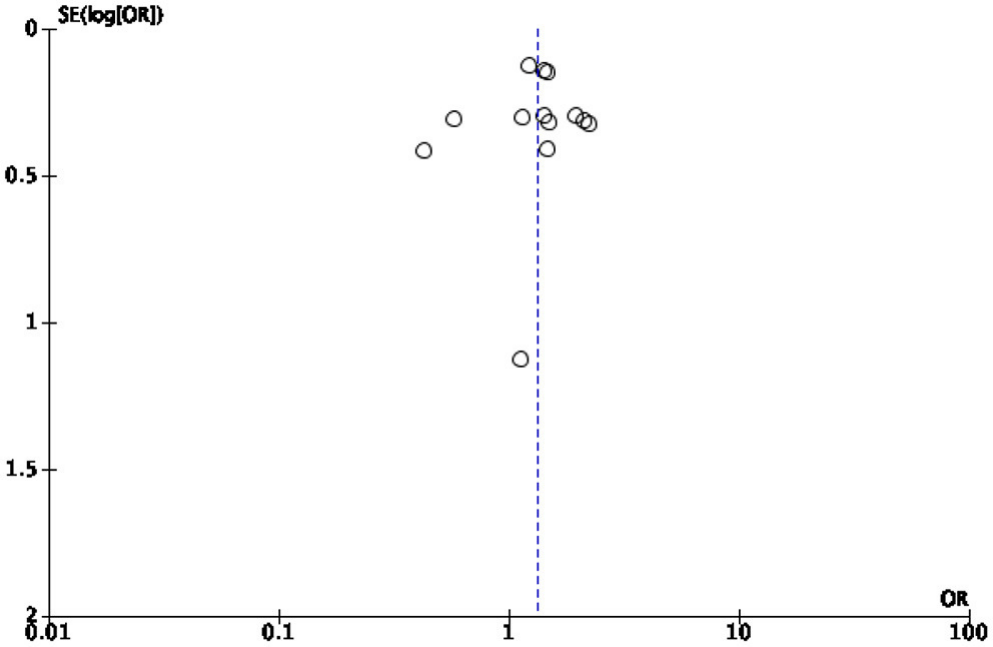
Publication bias for Morbidity.

This study presents several limitations mostly due to the high heterogeneity of the study population. A first limitation is the relatively small number of studies included in the synthesis given the large number of variables examined. Some of our analyses were based on a small (<5) number of studies, which is typically considered the minimal threshold for random-effects meta-analyses to maintain to maintain statistical power. In particular, the quantification of hospital admissions could not be continued because the meta-analysis showed that too few studies shed light on the topic of “hospitalizations” to quantify them in a statistically significant way. Secondly, no review protocol was published prior to the start of our analysis. Thirdly, we included in the synthesis only studies written in one of the languages spoken by the research team. This limitation had no effect on the final synthesis as all retrieved studies were written in English. Finally, some of the studies we analyzed appeared to be subject to a risk of bias. To minimize this risk, we implemented several bias assessments, especially a risk of bias and publication bias assessment. The results indicate that most studies under review (39) were assessed as being of high and moderate risk of bias, while 10 studies were assessed as having a low risk of bias. Publication bias assessment was conducted to assess small study effects via funnel plot (61, 62). In case of publication bias, the results of smaller studies are spread widely, due to lower precision, and asymmetrically around the average estimate compared to the results of larger studies. This asymmetry is suggestive of missing studies. In the absence of publication bias, individual study results are more evenly distributed around the pooled estimate (23, 62, 63). However, caution should be exercised when interpreting funnel plots especially when the number of included studies is smaller than 10 (25). In our cases, the funnel plots for diabetes related mortality and morbidity (Figures 4, 5, respectively) are hard to interpret.

In spite of these limitations, our study provided a systematic synthesis of digital measurements that can be predictive of mortality, morbidity, and hospitalizations among older adults. Our findings identify a number of digitally measurable physiological parameters that can serve as proxies for the worsening of an older person's health. This is information is critical to evaluate the current promises and challenges of digital health technologies in the care and health promising of older people, especially in the context of telemedicine and assisted living. Furthermore, this information can inform evidence-based decision making in the context of digital health and gerontechnology.

## Discussion

Our results identified the following predictors of mortality: diabetes, decreased BMI, arrhythmias, slower walking speed, and insufficient physical activity. Hypertension, diabetes and decreased BMI were also identified as significant predictors of morbidity, while frailty, pulmonary comorbidity, obesity, pain, fatigue, and fever were identified as significant predictors of hospital admission or readmission. Overall, our results show that personal digital health technologies that can adequately measure the above parameters have the potential to improve health outcomes for older people. This investigation is a prerequisite for the design, development, and deployment of personal digital health technologies that can effectively measure the most informative parameters and thereby leverage that information to enhance health outcomes within the older population segment. Our analysis indicates that a variety of health parameters, such as hemodynamic, respiratory, kinetic parameters, BMI, and diabetes, which are potentially collectable using personal digital technologies can be effectively used to predict and improve the health outcomes of older people aged 65 or older. Further, digital technologies such as blood pressure monitors, pulse oximeters and sensors for the measurement of heart and respiratory rate, blood glucose meters for diabetes, height-weight monitors for BMI, movement sensors, accelerometers, pedometers for physical activity parameters, dynamometers for muscle strength, spirometers and hand-held echocardiogram can be efficiently incorporated in routine-care of older people, since they are correlated with survival or mortality, respectively. All the digitally measurable predictors of morbidity pertained to parameters that can be managed remotely using personal digital health technology. Our results suggest that the incorporation of blood pressure monitors, of blood glucose monitors, of digital height-weight monitors, of movement sensors and stopwatches aiming to measure physical activity and gait speed as well as the incorporation of hand-held echocardiogram in routine care of older people can efficiently contribute to health maintenance and to the protection from adverse health conditions. Since the purpose of the current research was to provide a synthesis of the new technologies that can be used to measure risk factors of morbidity, we did not distinguish morbid conditions regarding their pathogenesis.

These results are consistent with previous studies that revealed positive correlations between specific technologies and health outcomes. For example, the use of remote digital arrhythmia monitoring has been observed to have an impact on medical care regarding hospitalization rates and effects on morbidity and mortality (64, 65). The systematic and meta-analytic nature of our study, however, allows contextualizing this evidence against a broader technological and medical context, comparing different data sources and thereby achieving more solid and generalizable knowledge. Some of the associations revealed by our study may appear prima facie counter-intuitive. One of them is the fact that obesity is positively associated with survival (OR 0.70; CI 0.56, 0.87; 6 studies) in older adults. However, this so-called “obesity paradox” appears to be well-known. Among others, Abramowitz et al. (66). report that numerous studies over the past two decades have shown a body-mass index (BMI) in the normal range is associated with the lowest risk of death. Other large cohort studies in various populations have reached different conclusions, demonstrating a survival benefit for overweight or even obesity, which has been interpreted by many as a causal relationship (66). Although obesity has been associated with a higher risk for cardiovascular and peripheral diseases and also for different types of cancer, previous studies have found that, in cases of acute decompensation or chronic hypertensive disease, type 2 diabetes, chronic kidney disease, or metastatic cancer, obese people in the older population segments tend to live longer (67–69), suggesting that obesity-induced health outcomes depend on variables such as age (68). Although for younger patients obesity is a risk factor for a higher mortality, in older patients it can become protective due to greater reserve for the fight against a disease. In the elderly, recent studies indicate that obesity is associated with a lower mortality risk (70, 71). These findings could be possibly explained by the fact that many previous studies were retrospective analyses which did not examine obesity as primary outcome and did not control for potential confounders that could influence the outcome, such as the presence of specific chronic conditions (69, 72). Further, current data are compatible with the view that not obesity but BMI changes are the primary factor which requires continuous monitoring in the old age as losing weight with age is generally associated with worse outcomes. Nonetheless, the possibility of this “obesity paradox” continues to be debated in the literature and is of great public health importance, not least because of the message communicated to the public (66). Another counter-intuitive result is that hypertension does not appear to be a significant predictor of mortality of people aged 65+. However, the little effect of blood pressure values on mortality risk is not surprising. As part of their treatment for stroke and CHD, many of the individuals were under treatment with agents to decrease triglyceride or lipid levels. It is possible that inclusion of categorical diagnostic information for hypertension and lipid treatment could have improved the prediction model. Unfortunately, these data are not currently available to us. However, we will note that hypertension exerts its deadly effects through CHD and stroke, so it is possible that some if not most of all the variance with respect to death are being captured by those variables (49).

## Conclusions

Our meta-analysis has systematically reviewed and compared 43 studies. Our results identified the following predictors of mortality for people aged 65 years or older: diabetes, reduced BMI, arrhythmias, slower walking speed, and insufficient physical activity. Hypertension, diabetes and decreased BMI were also identified as significant predictors of morbidity. Overall, our results show that digital health technologies that can adequately measure the above parameters have the potential to improve health outcomes for older people. This information is essential to develop digital health technologies for older people that could improve their overall health and well-being.

## Data Availability Statement

The original contributions presented in the study are included in the article/Supplementary Materials, further inquiries can be directed to the corresponding author/s.

## Author Contributions

SD developed the methodology, collected and analyzed the data, and drafted the manuscript. ARa conceived of the study, reviewed the data, and contributed to the manuscript. CH and MW reviewed the data and contributed to the manuscript. ARu obtained the funding, conceived of the study, and contributed to the manuscript. AS and NP analyzed the data and contributed to the manuscript. RK contributed to the study design and to the manuscript. MI obtained funding, conceived of the study, developed the methodology, reviewed the data, and drafted the manuscript. All authors approve the final version of this manuscript.

## Conflict of Interest

ARa, ARu, and MW were employed by Clever.Care AG. The authors declare that the research was conducted in the absence of any commercial or financial relationships that could be construed as a potential conflict of interest. This study was funded by Innosuisse- Swiss Innovation Agency, Grant Number: 40158.1 INNO-ICT. This funding scheme is purposively designed to facilitate and promote collaboration between academia and private companies.
