# Supplementary material for: Digital Predictors of Morbidity, Hospitalization, and Mortality Among Older Adults: A Systematic Review and Meta-Analysis
Source: Front Digit Health. 2021 Feb 4;2:602093. doi: 10.3389/fdgth.2020.602093 (PMC8521803; doi:10.3389/fdgth.2020.602093)
Supplement: Supplementary file 2 [file Data_Sheet_2.docx]

Annex 1. Keyword-chains formulated for each of the 3 remaining databases searched

Web of Science

**TOPIC:** (digital* measure*) *OR* **TOPIC:** (digital* obtain*) *OR* **TOPIC:** (digital* acquir*) *OR* **TOPIC:** (digital intervention*) *OR* **TOPIC:** (computer* intervention*) *OR* **TOPIC:** (digital technolog*) *OR* **TOPIC:** (emerging technolog*) *OR* **TOPIC:** (smartphone*) *OR* **TOPIC:** (smart home environment) *OR* **TOPIC:** (smart care environment) *OR* **TOPIC:** (mobile*) *OR* **TOPIC:** (robot*) *OR* **TOPIC:** (ambient assisted living) *OR* **TOPIC:** (intelligent medical assistant*) *OR* **TOPIC:** (artificial intelligence assistant*) *OR* **TOPIC:** (personal digital assistant*) *OR* **TOPIC:** (digital*) *OR* **TOPIC:** (sensor*) *OR* **TOPIC:** (wearable) *OR* **TOPIC:** (wireless)

AND

**TOPIC:** (old) *OR* **TOPIC:** (older) *OR* **TOPIC:** (senior*) *OR* **TOPIC:** (aged) *OR* **TOPIC:** (aging) *OR* **TOPIC:** (elder*)

AND

**TOPIC:** (predictor*) *OR* **TOPIC:** (indicator*) *OR* **TOPIC:** (discriminator*) *OR* **TOPIC:** (determinant*)

AND

**TOPIC:** (hospitalisation) *OR* **TOPIC:** (morbidity) *OR* **TOPIC:** (mortality)

Embase

'digital* measure*':ti,ab,kw OR 'digital* obtain*':ti,ab,kw OR 'digital* acquir*':ti,ab,kw OR 'digital intervention*':ti,ab,kw OR 'computer* intervention*':ti,ab,kw OR 'digital technolog*':ti,ab,kw OR 'emerging technolog*':ti,ab,kw OR smartphone*:ti,ab,kw OR 'smart home environment':ti,ab,kw OR 'smart care environment':ti,ab,kw OR mobile*:ti,ab,kw OR robot*:ti,ab,kw OR 'ambient assisted living':ti,ab,kw OR 'intelligent medical assistant*':ti,ab,kw OR 'artificial intelligence assistant*':ti,ab,kw OR 'personal digital assistant*':ti,ab,kw OR sensor*:ti,ab,kw OR wearable:ti,ab,kw OR wireless:ti,ab,kw

AND

old:ti,ab,kw OR older:ti,ab,kw senior*:ti,ab,kw OR aged:ti,ab,kw OR aging:ti,ab,kw OR elder*:ti,ab,kw

AND

predictor*:ti,ab,kw OR indicator*:ti,ab,kw OR descriminator*:ti,ab,kw OR determinant*:ti,ab,kw

AND

mortality:ti,ab,kw OR morbidity:ti,ab,kw OR hospitalisation:ti,ab,kw

PsycINFO

((noft(digital* measure*) OR noft(digital* obtain*) OR noft(technolog*) OR noft(smartphone*) OR noft(robot*) OR noft(mobile*) OR noft(digital*) OR noft(sensor*) OR noft(wearable) OR noft(wireless)) AND PEER(yes)) AND ((noft(old) OR noft(older) OR noft(senior*) OR noft(aged) OR noft(aging) OR noft(elder*)) AND PEER(yes)) AND ((noft(predictor*) OR noft(indicator*) OR noft(discriminator*) OR noft(determinant*)) AND PEER(yes)) AND ((noft(hospitalisation) OR noft(morbidity) OR noft(mortality)) AND PEER(yes))
